# Genetic Divergence between Freshwater and Marine Morphs of Alewife (*Alosa pseudoharengus*): A ‘Next-Generation’ Sequencing Analysis

**DOI:** 10.1371/journal.pone.0031803

**Published:** 2012-03-15

**Authors:** Sergiusz Czesny, John Epifanio, Pawel Michalak

**Affiliations:** 1 Lake Michigan Biological Station, Illinois Natural History Survey, University of Illinois, Zion, Illinois, United States of America; 2 Illinois Natural History Survey, University of Illinois, Champaign, Illinois, United States of America; 3 Virginia Bioinformatics Institute and Department of Biological Sciences, Virginia Tech, Blacksburg, Virginia, United States of America; Ecole Normale Supérieure de Lyon, France

## Abstract

Alewife *Alosa pseudoharengus*, a small clupeid fish native to Atlantic Ocean, has recently (∼150 years ago) invaded the North American Great Lakes and despite challenges of freshwater environment its populations exploded and disrupted local food web structures. This range expansion has been accompanied by dramatic changes at all levels of organization. Growth rates, size at maturation, or fecundity are only a few of the most distinct morphological and life history traits that contrast the two alewife morphs. A question arises to what extent these rapidly evolving differences between marine and freshwater varieties result from regulatory (including phenotypic plasticity) or structural mutations. To gain insights into expression changes and sequence divergence between marine and freshwater alewives, we sequenced transcriptomes of individuals from Lake Michigan and Atlantic Ocean. Population specific single nucleotide polymorphisms were rare but interestingly occurred in sequences of genes that also tended to show large differences in expression. Our results show that the striking phenotypic divergence between anadromous and lake alewives can be attributed to massive regulatory modifications rather than coding changes.

## Introduction

Evolutionary processes are most frequently discussed in the context of geological time scale and species divergence associated with adaptations is seldom noticeable on a shorter temporal range. Anthropogenic activity, however, becomes a potent force that often accelerates progression of otherwise slow evolutionary change, via either direct artificial selection or indirect environmental transformations. A striking example of evolutionary change indirectly induced by the human footprint can be found in the recent expansion of natural range by a small clupeid fish, alewife *Alosa pseudoharengus*. Alewife, native to the North American Atlantic coast (from North Carolina to Labrador), invaded all five Laurentian Great Lakes between 1860 and 1955 [1]. They first made their way into Lake Ontario via the Erie Canal during 1860's and progressed through the Welland Canal to Lake Erie bypassing the natural barrier of Niagara Falls between 1913 and 1932 [1]. Then, alewife successively expanded to Lakes Huron, Michigan, and Superior completing their basinwide expansion by mid-1950.

**Figure 1:**
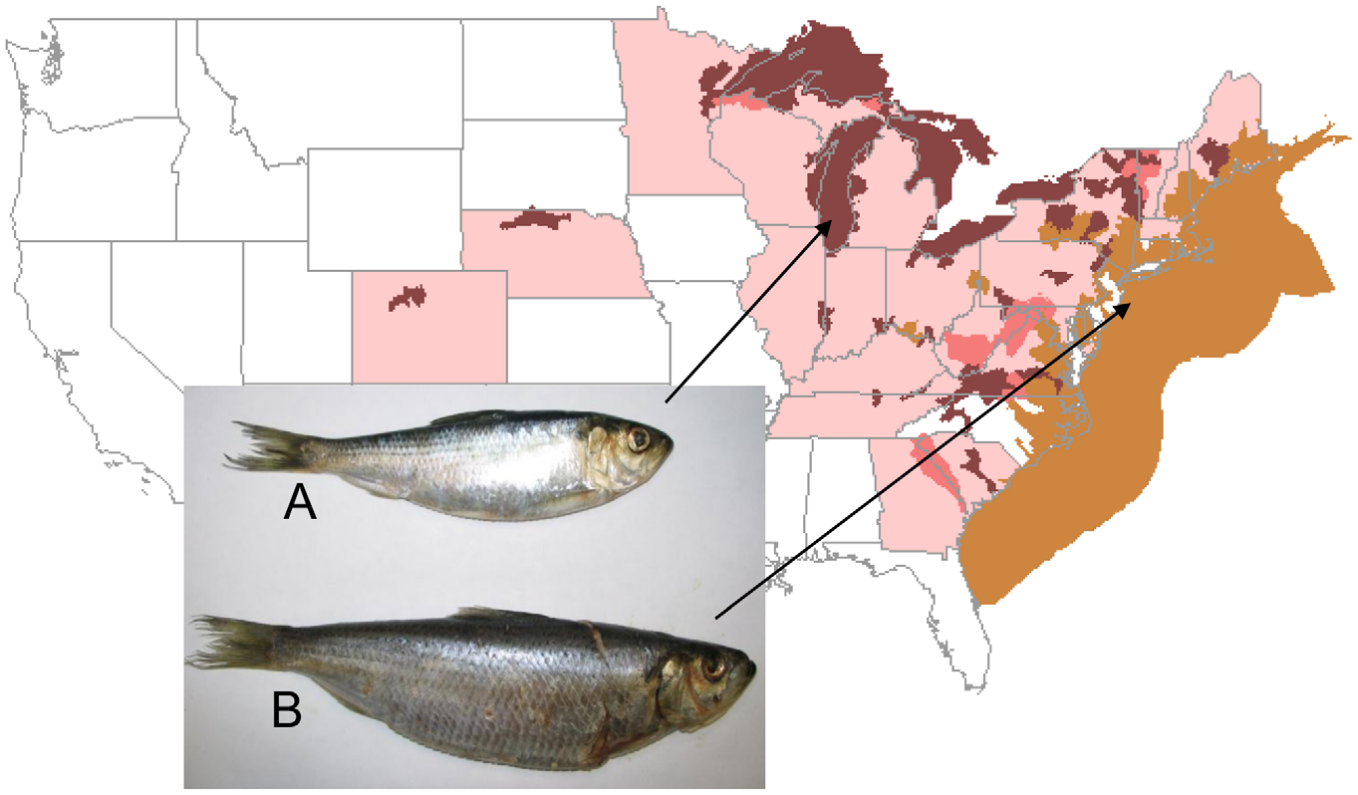
North American distribution of native (brown) and non-native (maroon and pink) ranges of alewife (*Alosa pseudoharengus*) along with the comparison of same-age individuals collected from freshwater Lake Michigan (A) and Atlantic Ocean (B). Arrows approximate sampling locations. Map is a courtesy of United States Geological Survey.

Divergent adaptations to freshwater versus marine environment have resulted in perhaps the most rapid morphological and life-history changes ever observed for a free-living vertebrate (Figure 1). Landlocked (freshwater resident of the Great Lakes) alewives mature earlier in life, have slower adult growth, smaller size at maturation, and reduced fecundity relative to anadromous (i.e., spawning in freshwater but spending most of their life in the ocean) alewives [2], [3]. It is unclear to what extent these changes are genetic adaptations, epigenetic modifications or mere phenotypic plasticity. It is expected, however, that the marine-freshwater environment shift immediately triggers cascades of dramatic regulatory responses and imposes very strong selection on landlocked populations over the long term. Alewife's ecological success and unusual adaptive ability offer a new intriguing model system to investigate regulatory as well as coding genetic innovations underlying rapid phenotypic adaptations.

The problem of adaptive genetics has been publicized by a recent “cis-regulatory” (evo-devo) versus “structural (coding) mutation” debate [4], [5]. Evo-devo side emphasizes significance of mutations in *cis* regions and regulatory rather than coding changes as a source of evolutionary novelties. Examples are provided by altered expression patterns of such genes as *Pitx1* and *Bmp4* underlying morphological changes in pelvic structure in sticklebacks [6], [7] and beak size in Darwin's finches [8], respectively. The other side of the debate points to a large body of evidence for structural changes in protein-coding sequences and their central role in evolution on one hand and the paucity of unequivocal evidence for evolutionarily significant regulatory changes on the other [4]. However stimulating this debate might be, it unfortunately also seems to oversimplify the complexity of adaptive processes. There is little doubt that both cis-regulatory and structural-coding changes shape adaptive traits and the question is more about their relative contributions rather than ruling out one or another. It must also be remembered that amino acid and regulatory changes are often inseparable as structural protein alterations may modulate expression (e.g. [9], [10]). Conversely, transcription seems to be capable of influencing mutational frequency as well. Increased mutations have been observed in highly transcribed regions in such diverse organisms as *Escherichia coli* [11], [12], yeast [13], and humans [14]. Epigenetic pathways along with their intricate interactions with environment, genome regulation and mutagenicity [15] add another, poorly known layer to adaptations. Additional dimension to genetics of adaptation is produced by phenotypic plasticity that in response to environmental challenges may expose hidden genetic variation to selection [16], [17].

Next generation sequencing of entire transcriptomes provides simultaneous information about sequence polymorphism and gene expression levels and can thus be useful in determining the prevalence of sequence and regulation divergence. Here, we sequenced gill-derived transcripts to get insights into expression changes and sequence divergence between marine and freshwater alewives. We hypothesized that (1) gills as the central respiratory and osmotic organ will be informative of transcriptional reprogramming required for transition between marine and freshwater environments, and that (2) mitochondrial genes with their higher mutation rate and essential role in transport chain will be good candidate sequences for molecular divergence between the two alewife morphs.

In addition to being a spectacular example of rapid and parallel evolution, invasive alewives with their complex and broad interactions with native fauna and tremendous plasticity in adaptations to new environments have status of a key player in their relatively new ecosystem (Great Lakes). Alewife population levels vary greatly among the individual Great Lakes with relatively low densities in Lakes Superior, Erie, and Huron and higher densities in Lakes Michigan and Ontario. Alewife has proven to impact native and naturalized fish species in a number of ways including competition for food, physical displacement from spawning habitats, source of nutritional stress in predators, and predation on eggs and larvae of other fishes.

## Methods

Seven 4 year old females of *Alosa pseudoharengus* were included in this study; four from Atlantic Ocean (AO) and three from Lake Michigan (LM). Fish from AO were obtained through NEFSC Ecosystem Surveys Branch in the fall of 2008. Samples were collected on 10/24/2008 from the mid-water trawl (depth 180m) conducted 108 miles due East off Portsmouth, NH (LAT 43.110879; LON -68.373058). Fish from Lake Michigan were collected on 11/24/2008 in bottom trawl 6 miles NE off Point Beach State Park, WI (LAT 44.274167, LON -87.410833). Immediately upon catching, 2–3 gill rakers were dissected from anesthetized individuals and preserved in RNAlater (Ambion/ABI). Fish were than frozen for age verification using otolith annual ring count.

Total RNA was extracted from gill-rakes using the RiboPure isolation kit (Ambion/ABI). RNA samples were quantified with the Agilent 2100 Bioanalyzer (Agilent Technologies). Double-stranded cDNA was produced using 1 µg of total RNA for each sample and the MINT cDNA synthesis kit (Evrogen). The ds cDNA quantity and quality were assessed with the Qubit fluorometer (Invitrogen) and the Quant-iT dsDNA HS Assay Kit. GS FLX Titanium (Roche) amplicon libraries were constructed separately from each individual and sequenced at Duke IGSP Sequencing Core Facility. The libraries were not normalized to allow quantification of relative transcript levels between AO and LM individuals.

NGen 2.0 (DNASTAR) was used to trim read ends for remaining adapter and poly A/T tail traces and make a *de novo* assembly (Expected coverage = 20; Specific match threshold = 90; Specific match size = 15; MM penalty = 20; Max gap = 15). SNPs were detected using SeqMan (DNASTAR) and to account for sequencing errors. Only sites polymorphic in 25–75% raw reads realigned against the assembly were included. Custom PERL scripts were used to parse NGen output for group-exclusive (AO vs. LM) SNPs. Blast2Go with default parameters was used to annotate all contigs.

Raw reads were normalized and quantified against the NGen assembly using ArrayStar (DNASTAR). The sensitivity of RNA-Seq is a function of both molar concentration and transcript length. Hence, we quantified transcript levels in reads per kilobase of exon per million mapped reads (RPKM-normalized). The RPKM measure reflects the molar concentration of a transcript in the starting sample by normalizing for RNA length and for the total read number in the measurement [18]. To test for differential expression, we used moderated t-test with the Benjamini-Hochberg correction for false discovery rate (FDR).

## Results

A total of 817,488 reads were produced from the seven cDNA samples (ranging between 51,697 to 246,125 per sample), with an average read length of 187 bp (Table S1). The reads were used for a *de novo* assembly resulting in 52,932 contigs with an average length of 351 bp and average depth of 10 sequences (ranging from 2 to 4,384). A total of 572,065 reads (70%) were incorporated into the assembly. The remaining 245,423 (unassembled) reads were singletons that tended to be significantly shorter (average 172 bp) from assembled reads (average 236 bp).

**Figure 2:**
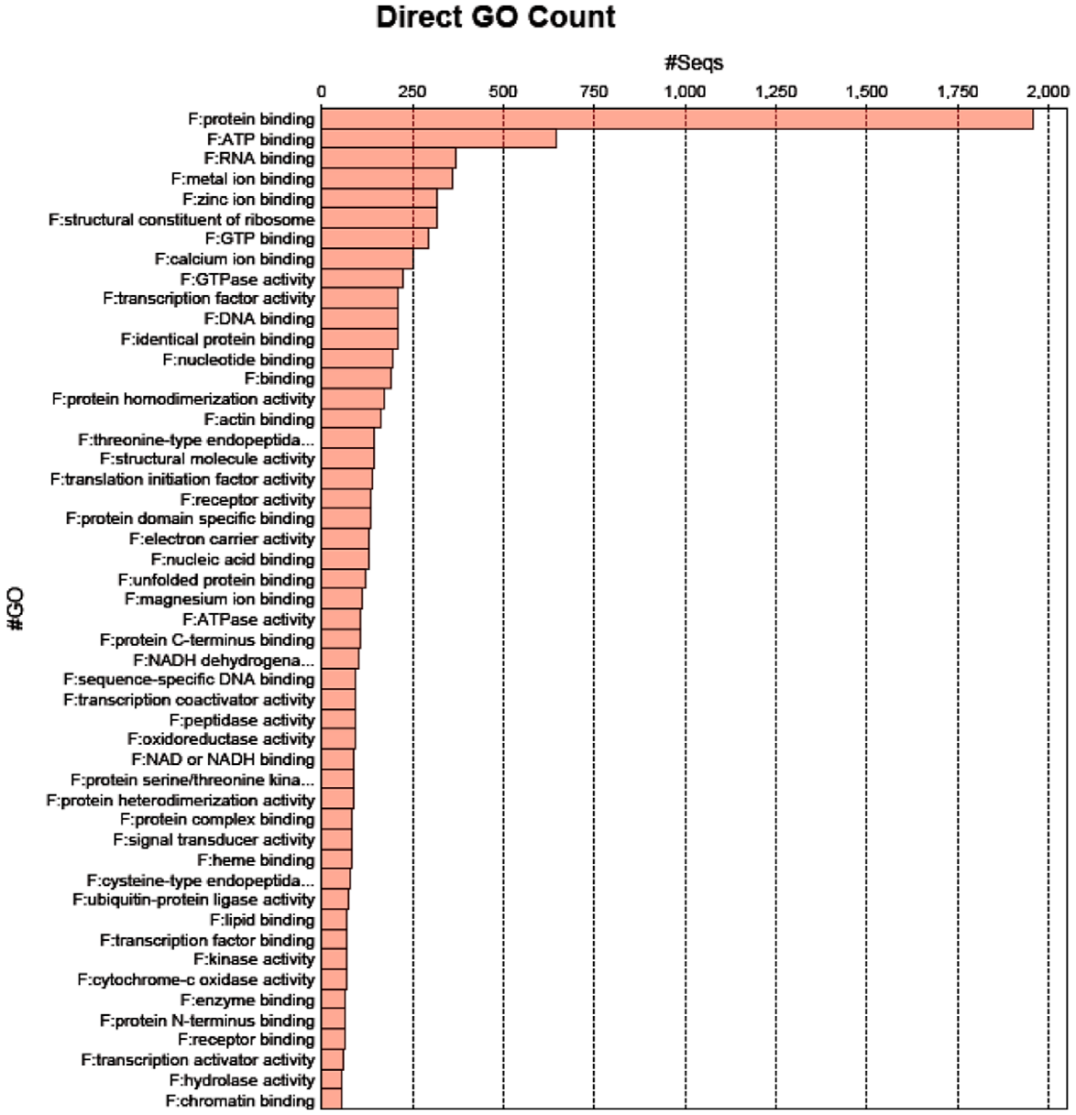
Most populated GO classes (Molecular Function) in the transcriptome assembly.

The contigs were subsequently analyzed with Blast2Go for similarities to sequences in public databases. Blast-assigned functional annotations were found for a total of 26,021 (49%) contigs, with around 5,000 representing enzymatic activity, 2,100 ribosomal proteins, 1,600 mitochondrial and respiratory functions, and 530 sequences related to Major Histocompatibility Complex (MHC). The following Gene Ontology (GO) molecular functions were most frequently represented among annotated contigs (Figure 2): protein binding (26%), ATP binding (9%), RNA binding (5%), metal ion binding (5%), and zinc ion binding (4%). Among most abundant GO biological processes, there were oxidation reduction (6%), auxin biosynthetic process (5%), signal transduction (3%), transport (3%), and proteolysis (3%). Both cytoplasm/cytosol and nucleus GO cytological components were highly represented, 36% and 15% respectively, along with ‘integral to membrane’ (12%), and mitochondrion (7%) classes. Most Blast2Go hits were from *Danio rerio* (8,282), *Salmo salar* (3,469), *Tetraodon nigroviridis* (1,633), *Osmerus mordax* (1,157), *Oncorhynchus mykiss* (1,131), *Anoplopoma fimbria* (497), and *Esox lucius* (459). These proportions confound the number of sequences available in NCBI's databases for each species, their annotation quality, and taxonomic distances between species (*Osmerus* and *Anoplopoma* are overrepresented given the number of sequences available in NCBI).

**Figure 3:**
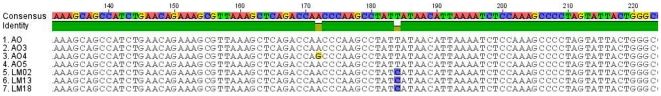
An example of alignment with a group specific SNP (16S ribosomal RNA gene).

A total of 439,311 putative SNPs were found. Since 454 reads are known to contain more sequencing errors than the standard sequencing technology [19], we filtered putative SNPs occurring in 25–75% sequences per contig. There were 6,835 such polymorphic sites in 5,853 contigs. To identify group specific (i.e., LM or AO) SNPs, we further filtered only those sites that contained at least one group-exclusive SNP. We found only 22 group-specific SNPs (0.3%) in 13 putative loci (out of which 5 could not be annotated; Table 1). With only three exceptions (including a putative indel), all group-specific SNPs were synonymous (Figure 3). Ten group-specific SNPs were polymorphic in either AO or LM, with maximum two alternative alleles per locus out of which one allele was always shared with the other group. Consistent with the demographic scenario of AO as a genetic source for LM, AO retained more such polymorphic sites (8) than LM (2) but this difference was not statistically significant (Fisher's exact test, P = 0.069).

**Table 1:** List of AO- and LM-specific SNPs.

gene	SNPposition (bp)	AO SNP base	LM SNP base	depth	aa change
Cytochrome b	786 (CDS)	A,G	G	41	-
Cytochrome b	1,052 (CDS)	C	T	117	-
NADH dehydrogenase subunit 1	+285 (CDS)	T	C	109	-
16S ribosomal RNA	465 (rRNA)	A,G	A	446	-
16S ribosomal RNA	706 (rRNA)	T	C	625	-
Eukaryotic translation initiation factor 2	562 (CDS)^i^	G	A	18	-
Ribosomal protein L36	21 (3′UTR)	G	T	34	-
60S ribosomal protein L7	636 (CDS)^ii^	C,T	C	380	-
60S ribosomal protein L17 putative	113 (CDS)iii	-	A	74	?
60S ribosomal protein L17 putative	203 (CDS)iii	C	T,C	85	-
Claudin 30c	57 (CDS)^iv^	T	G	30	-
MHC class II beta antigen	326 (CDS)^i^	A,G	G	203	-
MHC class II beta antigen	332 (CDS)^i^	G	A	205	T,M → I
Thymosin beta-12 putative	4 (3′UTR)^i^	A,G	A	150	-
Thymosin beta-12 putative	6 (3′UTR)^i^	T,G	T	152	-
Annexin A1	499 (CDS)^ii^	C	T	76	-
Myeloperoxidase	2,051 (CDS)^iv^	G	A	355	-
Unknown, coding-like (contig # 05589)	-	A	A,T	56	L → H ?
Noncoding, near transmembrane protein 59-like	671 (from 3′ end)^v^	G	A	29	-
Unknown, noncoding (contig # 349)	-	G,T	G	113	-
Unknown, noncoding (contig # 349)	-	A,C	A	120	-
Unknown, noncoding (contig # 349)	-	G,T	T	120	-
Unknown, noncoding-like (contig # 34454)	-	T	C	42	-

i Reference: *Danio rerio* CDS;

ii Reference: *Osmerus mordax* CDS;

iii *Esox lucius* CDS;

iv Reference: *Ictalurus punctatus* CDS;

v G*allus gallus* (75% identity).

**Figure 4:**
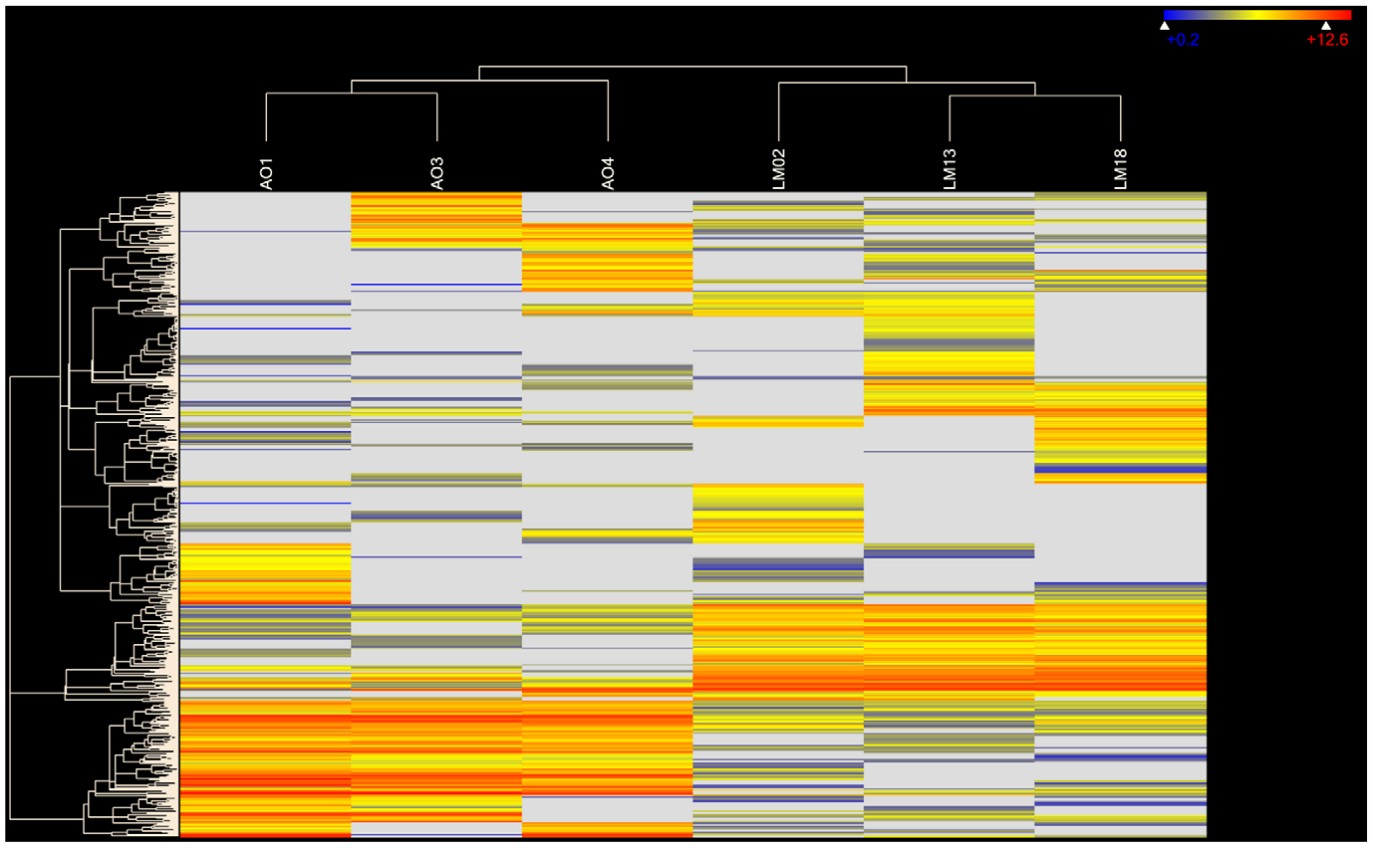
Hierarchical clustering of gene expression in alewives from the Atlantic (AO) and Lake Michigan (LM) populations. Red color reflects overexpression in LM and blue in AO (intermediate expression is in yellow; lack of expression – in white). A total of 621 genes differentially expressed, genes at 8-fold change were used and Euclidean distance metric with centroid (fast) linkage method was implemented.

We analyzed the total reads against the *de novo* assembly to identify contigs that were differentially expressed between AO and LM. One AO sample that produced a low number of reads (51,697), on average shorter (>100 bp) and worse quality than the remaining samples was excluded from this analysis. The quantification of RPKM-normalized read counts showed extensive expression differences between AO and LM (Figure 4). There were a total of 7,935 contigs (15%) differentially expressed at a 2-fold change level or greater. We then narrowed down the analysis to those 621 whose expression levels were at least 8-fold different. After the FDR adjustment (Benjamini Hochberg method), 29 of such contigs remained overexpressed and 23 underexpressed in LM relative to AO, and at the same time had functional annotations from the Blast2Go search (Tables 2 and S2). Mitochondrial 16S rRNA gene and putative intelectin 4 were among the most overexpressed genes in LM and AO, respectively. Various ribosomal protein genes (8) and keratin genes (5) seemed to be overrepresented among both up- and downregulated genes. Mitochondrial genes (*COI*, *cytb, NDUFA1*) involved in respiratory complexes were upregulated in LM, along with *cytochrome c oxidase subunit 4*, the terminal oxidase in mitochondrial electron transport, which is probably encoded in the nucleus (chromosome 20 in *H. sapiens*). Remarkably, four genes (31%) characterized by group-specific mutations, *18S rRNA*, *cytb,COI*, *NDUFA1*, and *Ribosomal protein L36* also belonged to the most misexpressed genes between LM and AO.

**Table 2:** Most differentially expressed genes between AO and LM (P-values are FDR-corrected).

functional description	fold change (LM/AO)	T-value	P-value
**Upregulation**			
16S ribosomal RNA gene	233.28	−14.95	4.24×10^−6^
pituitary homeobox 1 (Pitx1) putative	10.03	−7.05	0.0001
aquaporin-3a	54.90	−6.52	0.0003
type II keratin E3-like protein mRNA	13.11	−6.36	0.0004
claudin 8c (cldn8c) gene	8.70	−6.12	0.0005
type I cytokeratin, enveloping layer	11.78	−6.26	0.0007
selenoprotein Pa putative precursor	15.01	−5.44	0.001
transgelin putative	8.77	−5.93	0.001
tubulin like	11.13	−5.15	0.001
epithelial membrane protein 2 putative mRNA	17.47	−5.93	0.002
zona pellucida glycoprotein 2 preproprotein-like	18.25	−4.97	0.003
magnesium transporter nipa2	8.55	−4.61	0.003
selenoprotein P, plasma, 1a putative	11.56	−4.33	0.004
chaperonin containing TCP1, subunit 4 (delta)	8.16	−4.60	0.004
poly A binding protein, cytoplasmic 1 b (pabpc1b)	13.23	−4.28	0.004
phosphate carrier protein, mitochondrial precursor putative mRNA, pseudogene cds	8.57	−4.87	0.004
elongation factor 1 alpha (EF1-alpha)	17.65	−4.29	0.006
ribosomal protein L7	22.94	−3.96	0.006
type I keratin	9.82	−3.92	0.006
GTP binding protein 4 (gtpbp4)	8.19	−3.73	0.007
40S ribosomal protein S6 putative	20.53	−4.49	0.007
ferritin heavy polypeptide	9.91	−3.58	0.01
pre-mRNA-processing factor 6-like	8.57	−3.43	0.02
iron-sulfur cluster assembly enzyme ISCU	10.70	−3.30	0.02
ribosomal protein SA (rpsa)	9.35	−3.17	0.02
MHC class II antigen beta chain	8.10	−3.35	0.02
60S ribosomal protein L3 putative	13.51	−2.90	0.02
CCAAT/enhancer binding protein (C/EBP), beta	9.36	−3.07	0.03
eukaryotic translation elongation factor 1 beta 2 (eef1b2)	8.25	−2.77	0.04
Downregulation			
putative intelectin 4 (itln4)	299.79	13.14	9.43×10^−6^
glyceraldehyde 3-phosphate dehydrogenase isoform 2	66.71	9.69	2.36×10^−5^
NADH dehydrogenase subunit 1 gene	77.88	11.20	2.52×10^−5^
putative keratin	31.34	8.91	0.00004
cytochrome oxidase subunit I (COI)	34.03	7.75	5.36×10^−5^
phosphorylase kinase, delta (calm2b)	32.06	8.53	0.00013
MHC class II invariant chain-like protein	17.31	7.00	0.00021
guanine nucleotide-binding protein subunit beta-2-like 1	18.63	7.21	0.00034
cytochrome c oxidase subunit 4 isoform 2	17.60	6.05	0.0005
keratin 14-like	8.62	5.89	0.001
FBP32-like	16.43	5.32	0.002
ribosomal protein L36a	13.34	5.79	0.002
beta-actin 1	14.24	5.73	0.002
Complement C1q-like protein 2 (c1ql2)	51.47	4.56	0.003
C-C motif chemokine 25 precursor putative	11.86	4.66	0.004
hemoglobin subunit alpha putative	12.72	4.65	0.004
ribosomal protein L7	22.94	4.49	0.005
28S ribosomal RNA gene	30.99	3.92	0.01
ribosomal protein S8	12.11	3.86	0.01
C1q-like-protein	28.03	3.23	0.02
18S ribosomal RNA gene	20.86	3.09	0.02
ribosomal protein L10 (rpl10)	8.55	3.00	0.03
cytochrome b	14.12	2.81	0.04

## Discussion

Marine teleost fishes tend to lose water through osmosis and gain ions, primarily Na^+^ and Cl^−^, through diffusion, whereas freshwater fishes tend to gain water and loose ions [20]. Transition from marine to freshwater conditions thus poses dramatic challenges at multiple levels of organization. At the physiological level, salinity changes exert profound osmoregulatory responses, including modifications of ion and water transport and permeability in gill, kidney, and gastrointestinal tract tissues [21]. Diadromous fishes, or those that travel between salt and fresh water, possess the remarkable ability (known as euryhalinity) to compensate for the physiological perturbations caused by varying salinity. Of particular interest are populations of diadromous species that got landlocked and permanently challenged to adapt to freshwater conditions. Alewives, *Alosa pseudoharengus*, provide a very recent example of rapid phenotypic divergence between anadromous and landlocked populations [22].

We examined the extent to which the phenotypic divergence is accompanied by regulatory and nucleotide changes. As expected for such a recent isolation event, we found little DNA sequence divergence between studied populations. Among the eight annotated genes with population-specific single nucleotide variants, osmoregulatory functions were represented by claudin-30. Claudins are four-transmembrane domain proteins that form the main component of tight junction strands and create charge- and size-selective pores in the paracellular cells [23], [24]. Tight junctions form a continuous intercellular barrier that regulates the transport of water, small solutes, and immune cells. There are at least 24 claudins in mammalian genomes [25] and 56 in the puffer fish, *Fugu rubripes* [26].

Although the population-specific SNP in alewife's claudin did not produce an amino acid substitution, many ‘synonymous’ mutations have been known to affect gene expression levels via such diverse mechanisms as mRNA processing, export, and stability, translation initiation and elongation, as well as protein folding [27]. In transgenic experiments, the use of a particular synonymous codon can increase the expression of a transgene by more than 1,000-fold [28]. Gill expression of claudins 27, 28a and 30 tends to be increased in freshwater habitats, as compared to sea water, in several teleosts [21], [29], [30], [31]. Consistent with this pattern, we detected highly elevated levels of claudin-8c transcripts in alewives from Lake Michigan (Table 2).

In addition to claudins and ion-transporters, aquaporins, integral membrane proteins from a larger family of major intrinsic proteins, are central to water permeability of the gill epithelium. Aquaporin-3 (AQP3) tends to be downregulated in eels following fresh- to sea-water transfer [32], [33], presumably to facilitate water absorption [34], [35]. Sea basses (*Dicentrarchus labrax*) that inhabit the open sea and lagoons, but occasionally enter rivers, had increased levels of AQP3 level in gills after acclimation to fresh water [36]. In this study, AQP3 was one of the most upregulated genes in alewives from the lake. It must be noted, however, that long-term adaptations at the level of gene expression may differ from expression profiles obtained during physiological responses to acclimation.

There was a substantial contribution of mitochondrial genes to genetic differences between freshwater and Atlantic alewives, and it is tempting to speculate that changes in genes related to respiratory complexes (*cytb* and *NDUFA1*) may have been of adaptive value in the ecological transition. The large representation of mitochondrial genes among transcripts derived from the gill is not unexpected, given that gill epithelium contains mitochondria-rich cells known as chloride cells [37].

The transition from anadromous to resident freshwater environment likely exposes the organism to a new suite of immunological stimuli. We found SNPs (including a nonsynonymous substitution) in DAB, one of the MHC genes from class II, thymosine, and annexin A1. Class I and class II MHC genes encode proteins responsible for recognizing self- and not self-peptides and present them to the T lymphocytes, inducing the immune response in vertebrates [38]. Class I molecules consist of two different subunits with only one of them, the alpha chain, participates in the antigen presentation. Class II molecules consist of two subunits, alpha and beta, encoded by different genes but both responsible for the peptide presentation [39]. Thymosin peptides act as immuno-transmitters and play a role in T cell maturation [40], [41], whereas annexins are Ca^2+^-dependent phospholipid-binding proteins involved in many cellular processes, including regulation of the innate and adaptive immune systems [42].

Our results suggest that the striking phenotypic divergence between anadromous and lake alewives can be attributed to massive regulatory modifications rather than coding changes. Future studies involving, for example, ‘common garden’ breeding experiments of both freshwater and anadromous alewives will be required to assess to what extent the regulatory changes are due to ultimate as opposed to proximate causes. Interestingly, many (31%) morph-specific synonymous mutations occurred in genes that also showed differential expression between morphs, potentially indicating a causative link between nucleotide changes and expression levels. Sequence divergence was overall low but it was higher in anadromous individuals, consistent with the fact that the Atlantic populations were a demographic source of landlocked populations.

## Supporting Information

Table S1 Total number of reads per sample.
(PDF)

Table S2 Candidate genes differentially expressed between AO and LM. Expression levels of individual samples are shown (columns C- I), in addition to annotations (columns A–B), fold-changes (L), and significance tests (J–K).
(XLSX)
