# Supplementary material for: Genetic Divergence between Freshwater and Marine Morphs of Alewife (Alosa pseudoharengus): A ‘Next-Generation’ Sequencing Analysis
Source: PLoS One. 2012 Mar 15;7(3):e31803. doi: 10.1371/journal.pone.0031803 (PMC3305293; doi:10.1371/journal.pone.0031803)
Supplement: Table S1 — Total number of reads per sample. (PDF) [file pone.0031803.s001.pdf]

Sample	Reads ##
AO1	246,125
AO2	151,216
AO3	78,033
AO4	51,697
LM1	114,714
LM2	72,396
LM3	103,307
